# Protein lysine crotonylation: past, present, perspective

**DOI:** 10.1038/s41419-021-03987-z

**Published:** 2021-07-14

**Authors:** Gaoyue Jiang, Chunxia Li, Meng Lu, Kefeng Lu, Huihui Li

**Affiliations:** 1 West China Second University Hospital, State Key Laboratory of Biotherapy, and Key Laboratory of Birth Defects and Related Diseases of Women and Children, Ministry of Education, Sichuan University, 610041 Chengdu, China; 2 Department of Neurosurgery, State Key Laboratory of Biotherapy, West China Hospital, Sichuan University and The Research Units of West China, Chinese Academy of Medical Sciences, Chengdu, China

**Keywords:** Cell signalling, Post-translational modifications

## Abstract

Lysine crotonylation has been discovered in histone and non-histone proteins and found to be involved in diverse diseases and biological processes, such as neuropsychiatric disease, carcinogenesis, spermatogenesis, tissue injury, and inflammation. The unique carbon–carbon π-bond structure indicates that lysine crotonylation may use distinct regulatory mechanisms from the widely studied other types of lysine acylation. In this review, we discussed the regulation of lysine crotonylation by enzymatic and non-enzymatic mechanisms, the recognition of substrate proteins, the physiological functions of lysine crotonylation and its cross-talk with other types of modification. The tools and methods for prediction and detection of lysine crotonylation were also described.

## Introduction

**Fig. 1:**
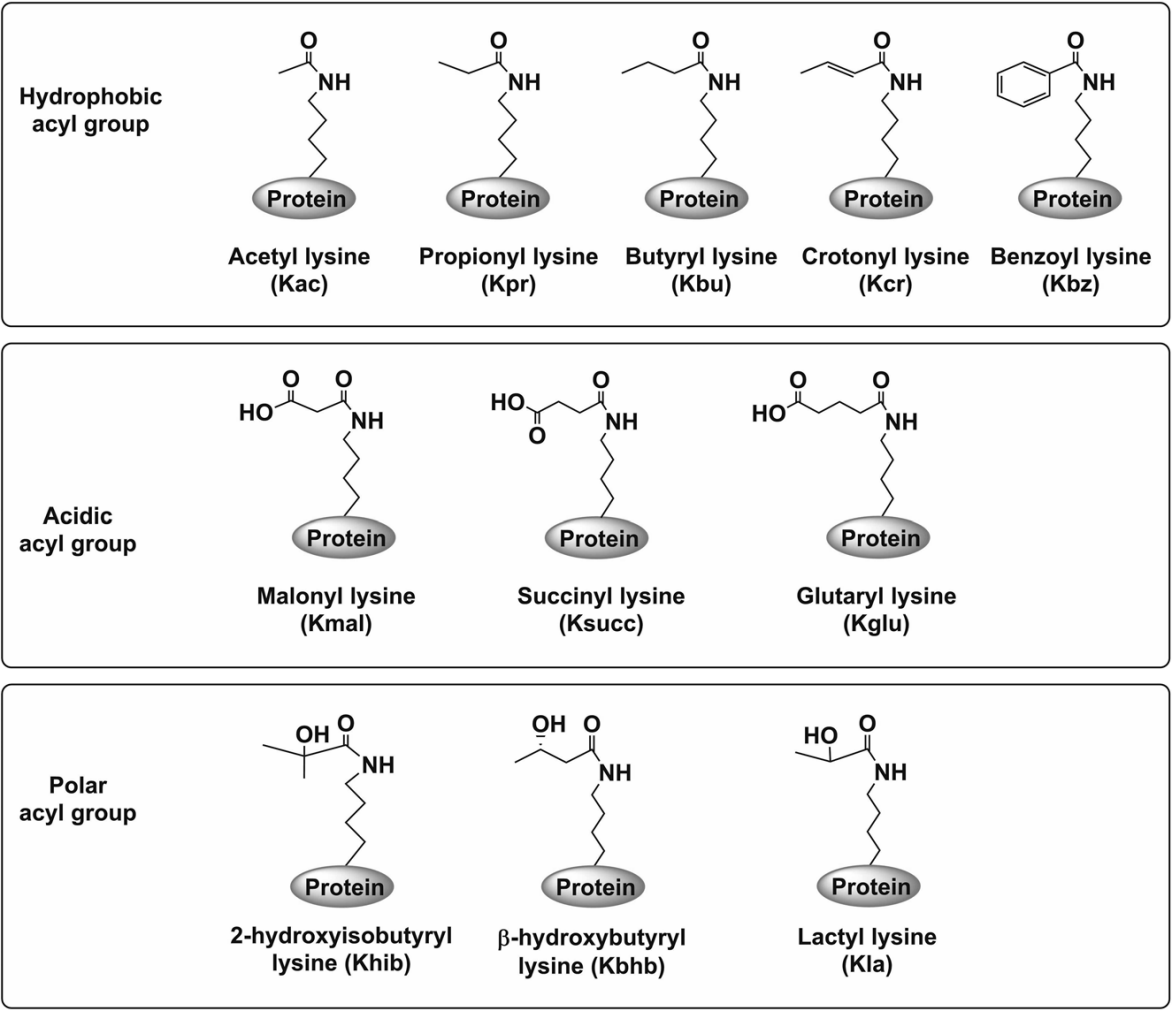
Chemical structures of lysine acylations. Based on their chemical properties, lysine acylations are classified into three groups: the hydrophobic acyl group that extends hydrocarbon chains, including Kac, Kpr, Kbu, Kbz, and Kcr; the polar acyl group includes Kbhb, Khib, and Kla that contain hydroxyl moiety to enable the modified lysine to form hydrogen bonds with other molecules; the acidic acyl group includes Kmal, Ksucc, and Kglu that alter the charge at the lysine residue from +1 to –1 at physiological pH [1].

Protein posttranslational modifications (PTMs) are important epigenetic regulatory mechanisms involved in diverse biological processes, such as DNA replication, transcription, cell differentiation, and organismal development. Dysregulation of PTMs is associated with a number of diseases, e.g., neuropsychiatric disease, carcinogenesis, and tissue injury [1]. Due to the development of high-resolution liquid chromatography with tandem mass spectrometry (LC–MS/MS) for the identification of PTMs, various lysine acylations including acetylation (Kac), butyrylation (Kbu), crotonylation (Kcr), propionylation (Kpr), malonylation (Kmal), glutarylation (Kglu), benzoylation (Kbz), 2-hydroxyisobutyrylation (Khib), β-hydroxybutyrylation (Kbhb), succinylation (Ksucc), and lactylation (Kla) have been identified [2, 3] (Fig. 1). These modifications influence protein structure and modulate their stability, localization, and activity [4]. Based on the chemical properties of lysine modification, acylations are classified into three groups (Fig. 1): the hydrophobic acyl group, the polar acyl group, and the acidic acyl group [1].

Crotonylation was initially identified on lysine residues in histones enriched in the promoter and enhancer regions in both human somatic and male germinal cells, indicating lysine crotonylation (Kcr) of histone may be an indicator of gene expression [5]. The histone Kcr was conserved from yeast to human [5]. Subsequently, non-histone crotonylation was identified to be particularly enriched in nuclear proteins involved in RNA processing, nucleic acid metabolism, and chromosome organization [6]. Later, more studies identified Kcr in non-histone proteins [7–9]. The crystal structure of the nucleosome containing crotonylated H3K122cr revealed that H3K122cr did not affect the overall nucleosome structure, but locally impeded the formation of water-mediated hydrogen bond with DNA backbone, weakened the histone–DNA association, thus favored the transcriptional activation [10]. Structurally, Kcr is four-carbon in length and the crotonyl modification contains a carbon–carbon (C–C) π-bond that results in a unique rigid planar conformation [1]. In this review, we will discuss the enzymatic and non-enzymatic regulation of crotonylation, the cellular and physiological functions of Kcr, the cross-talk between Kcr with other PTMs, and the prediction tools and detection methods for Kcr.

## Regulation mechanisms of Kcr

Protein lysine acylation such as Kcr, Ksucc, Kmal, Kglu, and Kbhb can be regulated by either enzymatic or non-enzymatic mechanisms [11]. Both serum and urine have been detected with trace amounts of short-chain fatty acid (SCFA) crotonate [12, 13]. Increased crotonate in colon lumen and serum caused elevated histone Kcr [14]. Supplementation with crotonate dramatically enhanced the levels of cellular crotonyl-CoA and histone Kcr [15]. Besides, treatment with crotonate significantly increased global Kcr [16], suggesting the abundance of crotonyl-CoA would be one of the main governing factors of Kcr.

**Fig. 2:**
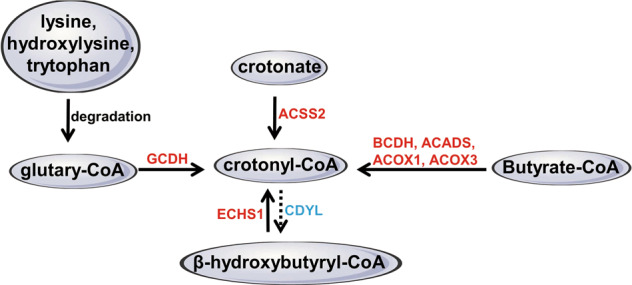
The generation of crotonyl-CoA. SCFAs such as crotonate can be metabolized to crotonyl-CoA by ACCS2 [15]. Besides, SCFA butyrate could be converted into butyryl-CoA through β-oxidation pathway, and further into crotonyl-CoA by BCDH [17]. ACADS and ACOX3 that catalyze conversion of butyryl-CoA to crotonyl-CoA during fatty acid oxidation were demonstrated to be as key crotonyl-CoA producers during endoderm differentiation [18]. In amino acid metabolism of lysine, hydroxylysine, and tryptophan, GCDH catalyzes the oxidation of glutaryl-CoA to crotonyl-CoA and CO_2_ [19, 20]. CDYL converts crotonyl-CoA into β-hydroxybutyryl-CoA [22].

The process converting crotonate into crotonyl-CoA was mediated by Acyl-CoA synthetase short chain family member 2 (ACSS2) [15]. Depletion of ACSS2 resulted in drop of cellular crotonyl-CoA and histone Kcr, indicating crotonate might be the endogenous source of crotonyl-CoA [15]. Besides, the SCFA butyrate through β-oxidation pathway was converted into glutaryl-CoA, and further into crotonyl-CoA by butyryl-CoA dehydrogenase (BCDH) [17]. Furthermore, the enzymes that catalyze conversion of butyryl-CoA to crotonyl-CoA during fatty acid oxidation, acyl-CoA dehydrogenase short chain (ACADS), and acyl-CoA oxidase (ACOX3) were key crotonyl-CoA producers during endoderm differentiation [18]. Deletion of ACADS or ACOX3 caused drop of intracellular crotonyl-CoA levels without affecting other acyl-CoAs [18]. During the amino acid metabolism of lysine, hydroxylysine and tryptophan, glutaryl-CoA dehydrogenase (GCDH) catalyzes the oxidation of glutaryl-CoA to crotonyl-CoA [19, 20]. The GCDH deficiency caused accumulation of glutarylcarnitine and neurotoxic glutaric acid, glutaryl-CoA and 3-hydroxyglutaric acid [21]. Furthermore, chromodomain Y-like (CDYL) was reported as a crotonyl-CoA hydratase that converts crotonyl-CoA into β-hydroxybutyryl-CoA and negatively regulates histone Kcr [22]. Therefore, these studies support the notion that crotonyl-CoA, crotonate, and butyrate may drive the occurrence of Kcr (Fig. 2).

**Fig. 3:**
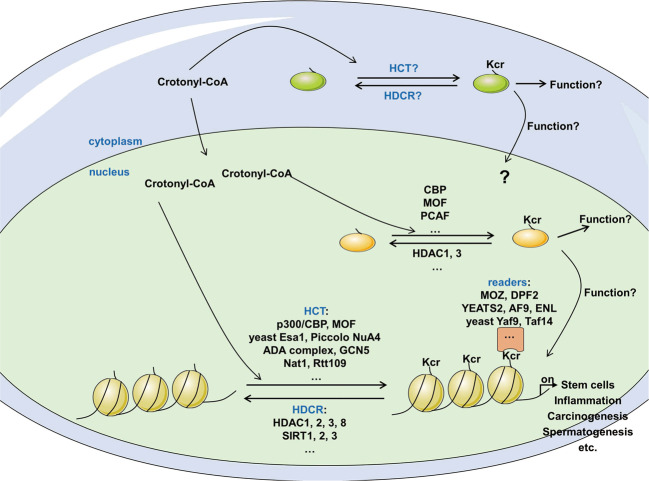
The modulation of protein crotonylation. Crotonylation has been identified on lysine residues in histone and non-histone proteins. Protein crotonylation was catalyzed by HCT such as p300/CBP [15, 24], MOF [24], and crotonyltransferases including CBP, MOF, and PCAF [9]. Modified crotonyl moiety could be removed by HDCRs HDAC1, 2, 3, 8 [16], SIRT1-3 [34] and decrotonylases HDAC1, 3 [9]. Furthermore, crotonylation acts as docking marks to recruit readers, e.g., DPF family proteins MOZ, DPF2 [38], YEATS domain proteins AF9, ENL [37] and YEATS2 [39].

Besides the regulation of Kcr by intracellular crotonyl-CoA levels, several recent studies have demonstrated enzyme-regulation on Kcr. The regulation of Kcr is a dynamic balance between the enzymatic activities of writer and eraser proteins that add and remove modification, respectively. The identification and characterization of writers and erasers is essential for classifying the regulatory mechanisms of protein crotonylation (Table 1, Fig. 3).

**Table 1:** writers, erasers and readers of Kcr.

	Family	Targets	Enzymes	Ref.
Writers	p300/CBP family	histone	p300, CBP	[15, 24]
		non-histone protein NPM1, DDX5	CBP	[9]
	MYST family	histone	MOF, yeast Esa1	[24]
			yeast Piccolo NuA4 complex	[29]
		non-histone protein NPM1	MOF	[9]
	GNAT family	histone	yeast Gcn5, Hat1	[26]
			yeast (ADA) complex	[29]
		non-histone protein NPM1, DDX5	PCAF	[9]
Erasers	HDAC I family	histone	HDAC3–NCoR1	[31]
			HDAC1, 2, 3, 8	[16]
			HDAC1, 2, 3	[14]
			HDAC1, 2	[32]
		non-histone protein NPM1	HDAC1, 3	[9]
	HDAC III family	histone	SIRT1, 2	[33]
			SIRT1, 2, 3	[34]
			SIRT1	[16]
Readers	YEATS domain family	histone	AF9, ENL, yeast Yaf9, Taf14	[37]
			YEATS2	[39]
			yeast Taf14	[26]
	DPF domain family		MOZ, DPF2	[38]
	bromodomain family		BRD9, TAF1 (but much weak than Kac)	[36]

### Kcr writers

Enzymes that catalyze modification are referred to as writers. However, crotonyl-specific writers have not been identified yet. Previously characterized histone acetyltransferases (HATs) were shown to have expanded histone crotonyltransferase (HCT) activities. Three major HAT families including p300/CREB-binding protein (p300/CBP), MYST, and GNAT (Gcn5-related N-acetyltrasferase) were characterized by their sequences and structures (Supplementary Fig. 1), and have been reported as HCTs that use crotonyl-CoA as substrate to catalyze Kcr [1].

The p300/CBP have both HAT and HCT activities, and p300-catalyzed histone Kcr can directly stimulate transcription [15]. A hydrophobic pocket, predicted to accommodate the aliphatic portion of remodeled acyl-CoA in the active site of p300, was observed in the crystal structures of p300 in complex with propionyl-CoA, crotonyl-CoA, or butyryl-CoA [23]. The size of the pocket and its aliphatic nature restrict against long-chain acyl-CoA variants and instead accommodate short-chain Acyl-CoA such as acetyl-CoA, propionyl-CoA, crotonyl-CoA, or butyryl-CoA without major structural rearrangements [23]. However, due to the restricted size of an aliphatic back pocket and a substrate-assisted rearrangement of the acyl-CoA chain, the acyltransferase activity of p300 gets weaker with increasing acyl-chain length [23]. Still, p300/CBP was considered to be the major HCT in mammalian cells [24], the p300/CBP mutants with deficient HAT but competent HCT activity substitute the endogenous CBP/p300 to enhance transcriptional activation [24]. Later, the global Kcr substrates regulated by p300 were involved in diverse cellular processes [25].

The MYST family proteins, human MOF, and its yeast homolog Esa1 were detected with a robust HCT activity on both histone H3 and H4 [24]. Deficiency of GNAT family proteins, Gcn5 and HAT1, caused considerably reduced H3K9cr levels in yeast [26]. However, neither human MOF, yeast Esa1 or yeast Gcn5 displayed any HCT activity in vitro [15, 24, 26], suggesting they may play HCT activities by forming complex [27, 28]. Indeed, Gcn5 with Ada2 and Ada3 formed ADA complex as a HCT for histone H3Kcr in yeast [29]. Besides, Esa1–Yng2–Epl1 complex was uncovered to function as histone H3 crotonyltransferase in yeast [29].

Recently, non-histone protein NPM1 was strongly crotonylated by CBP and MOF, and moderately crotonylated by p300/CBP-associated-factor (PCAF) [9]. However, crotonylation of non-histone protein DDX5 can only be catalyzed by CBP [9]. Non-histone proteins may have distinct HCTs because of their diverse locations.

### Kcr erasers

Enzymes that remove modification from specific residues in proteins are referred to as erasers. There are four groups of histone deacetylases (HDACs) [30] (Supplementary Fig. 2). Both class I and III HDACs were reported as histone decrotonaylases (HDCRs) [2].

HDAC3–NCoR1 complex was first reported to exhibit HDCR activity in vitro [31]. Treatment with histone deacetylase inhibitors vorinostat and apicidin inhibited the HDCR activity of HDAC3–NCoR1 [31]. Recently, class I HDACs were demonstrated as the major HDCRs in mammalian cells and displayed distinct site specificity from histone decrotonylation by class III HDAC (SIRT1) [16]. Given that class I HDACs exhibited a major HDCR activity while class II HDACs were deficient in HDCR activity, key residues of catalytic centers in class I and II HDACs were aligned and major differences were identified [16]. HDAC1 and HDAC3 mutants that lose HDAC but keep intact HDCR activity displayed a global transcriptional repression and diminished the promoter association with crotonylated histones [16]. Recently, HDAC1-3 regulated HDCR in colon in response to SCFA generated by microbiota of the gut [14]. Genetic deletion of HDAC1/2 in embryonic stem cells (ESCs) increased global histone crotonylation and resulted in 85% reduction in total HDCR activity [32].

The Class III HDACs, SIRT1-2 were acting as efficient HDACs [33]. By an optimized cross-linking assisted and stable isotope labeling of amino acids in cell culture-based protein identification approach to comprehensively profile erasers that recognize histone Kcr marks, human SIRT1-3 were identified as HDCRs [34]. The crystal structure of human SIRT3–H3K4cr complex was solved and the crotonyl-lysine of H3K4cr was located in a hydrophobic pocket of SIRT3 [34]. Residue His248 interacted with the crotonyl amide oxygen via hydrogen bonding and the phenyl ring of residue Phe180 aligned parallel to the planar crotonyl group and formed π–π stacking interaction with the C–C double bond of crotonyl-lysine [34]. Alignment of all sirtuins demonstrated that the residue Phe180 of SIRT3 is conserved in SIRT1-2, but not in other sirtuins, which may explain why SIRT4-7 were not identified as HDCRs [34]. However, the levels of histone crotonylation were higher in SIRT3 lacking cells, but not in those lacking SIRT1/2, suggesting endogenous SIRT3 as a main HDCRs [34].

Recently, crotonylated NPM1 was increased after a pan-HDAC inhibitor TSA treatment, suggesting HDACs may influence NPM1 crotonylation [9]. HDAC1 and HDAC3, but not HDAC2, decrotonylated NPM1, which can be reversed upon TSA treatment [9].

### Kcr readers

The level of Kcr could be influenced by the levels of intracellular crotonyl-CoA, and the ratio of crotonyl-CoA/acetyl-CoA, as well as the dynamic balance between crotonyltransferase and decrotonylase [2]. Thus, the function of Kcr modification in physiology and pathology may be dependent on the readers that recognize Kcr modification. For the well-studied histone Kac, three major families of readers have been characterized: bromodomain proteins, YEATS domain proteins, and double plant homeodomain finger (DPF) proteins [35] (Supplementary Fig. 3). Although a subset of bromodomain-containing proteins such as BRD9 and TAF1 were shown to recognize Kcr, their binding affinities are much weaker with crotonylated peptides than with acetylated peptides [36]. On contrary, DPF or YEATS domain proteins displayed preference for histone Kcr to other types of acylation [37, 38].

Recent studies demonstrated that the YEATS domain more favors Kcr than Kac [26, 37, 39]. Calorimetric titrations revealed that AF9 YEATS possesses a 2.4-fold binding enhancement for Kcr over Kac, and this favorable Kcr readout was conserved in human ENL, yeast Yaf9 and Taf14 [37]. The crystal structure of AF9 YEATS in complex with H3K9cr revealed that AF9 YEATS use the same Kac-binding aromatic sandwich cage for Kcr recognition, with only slight conformational changes of aromatic residues [37]. Besides the hydrogen bonding interactions, preferential binding to Kcr is notably contributed by π-aromatic interactions of the planar crotonylamide group with aromatic rings in the AF9 binding pocket [37]. By comparison between the crystal structures of BRD3-H3K18ac and AF9-H3K18cr, the mechanism of YEATS as preferential Kcr reader was displayed [36]. Kcr is too rigid for the reader pockets of most BRD proteins except for those that have a wider pocket, such as TAF1 [36]. However, the elongated and end-open reader packet of YEATS is ideal for interaction with acyl chains of Kcr. This unique mechanism [40] was also observed in human YEATS2 [39] and yeast Taf14 [26]. By targeting the π–π–π stacking in the aromatic ‘sandwich’ cage, a set of YEATS inhibitors were developed [41–46].

DPF domain proteins, including MOZ, MORF, and DPF1-3, were characterized as Kac readers [1]. Recently, the DPF domain was characterized as histone Kcr-preferential reader [38]. DPF domains of MOZ and DPF2 displayed 4 to 8-fold binding enhancement of Kcr over Kac [38]. The crystal structure of DPF domain of MOZ in complex with H3K14cr peptide revealed that a hydrophobic ‘dead-end’ pocket lacking aromatic sandwiching residues accommodated Kcr [38]. Notably, hydrophobic ‘dead-end’ pocket with selectivity for crotonylation was originated from intimate encapsulation and an amide-sensing hydrogen bonding network [38].

Therefore, the histone Kcr was recognized by π–π–π stacking mechanism of the YEATS domain and intimate hydrophobic ‘dead-end’ mechanism of the DPF domain.

## The functions of Kcr in physiology and pathology

Several recent studies have demonstrated that Kcr is implicated in various physiological processes [5, 15] (Fig. 3).

### DNA damage and repair

The level of H3K9cr exhibited rapid and transient decrease at DNA damage sites following DNA damage by exposing to laser-microirradiation, ionizing radiation, ultraviolet radiation or by treatment with etoposide damaging agents [47]. HDACs, but not SIRTs mediated the reduction in H3K9cr during DNA damage [47]. On the other hand, the level of RPA1 Kcr was upregulated upon DNA-damaging and was negatively regulated by CDYL1 [22]. The Kcr modification of RPA1 enhanced the interaction of RPA1 with single-stranded DNA and with components of resection machinery, and facilitated cell survival under DNA damage conditions [22]. Although the study indicated that CDYL reduced Kcr of RPA1, the possibility that RPA1 Kcr could be regulated by other factors such as HCTs and/or HDCRs could not be ruled out. These unidentified factors and CDYL together may contribute to the dynamics of RPA1 Kcr upon DNA-damaging.

### Neuropsychiatric disease

Under chronic social defeat stress and micro-defeat stress, lower level of histone Kcr was exhibited in the medial prefrontal cortex concurrent with selective upregulation of CDYL [48]. Furthermore, *Cdyl* expression in prelimbic cortex influenced the stress-induced depression-like behaviors in mice [48]. Subsequently, CDYL regulated stress-induced depression-like behaviors by inhibiting VGF nerve growth factor-mediated transcription, and this activity of CDYL was dependent on its dual hydratase function on histone Kcr and H3K27me3 at the VGF promoter [48]. Thus, CDYL-mediated reduction of histone Kcr played a critical role in regulating stress-induced depression [48]. Although lack of site-specific histone Kcr antibodies and mutants made it unable to specifically interrogate the function of Kcr, the observation that histone Kcr may affect major depressive disorders uncovered a possible regulatory mechanism that contributes to this neuropsychiatric disease.

In Alzheimer’s disease (AD), nuclear paraspeckle assembly transcript 1 (NEAT1), a long non-coding RNA, mediated the autoacetylation of p300, which altered the level of H3K27ac and H3K27cr and the transcription of endocytosis-related genes [49]. The low level of acetyl-CoA after NEAT1 inhibition caused decrease of H3K27ac and increase of H3K27cr [49]. This distinct alteration reveals the different roles of H3K27ac and H3K27cr in regulation of gene expression, which provides insight on the epigenetic regulatory mechanism of NEAT1 in AD pathology [49].

### Self-renewal and differentiation of stem cells

Histone Kcr was detected with much higher levels in mouse ESCs [16]. Induced HDAC1-VRPP mutant with intact HDCR but impaired HDAC activity caused marked reduction of histone Kcr and a drastic reduction of the ESC pluripotency factors, and an increase of endoderm, mesoderm, and ectoderm markers [16]. Thus, enriched histone Kcr was required for self-renewal of ESCs [16]. Recently, an enrichment of both H3K18ac and H3K18cr at bivalent genes upon deletion of HDAC1-2 in ESCs was observed [32], suggesting a role of HDAC1-2 in controlling the developmentally regulated genes prior to ES cell differentiation. Consistently, top 10% of genes enriched for either H3K18cr or H3K18ac upon HDAC1-2 deletion were functional in embryonic morphogenesis and embryo development [32].

Sufficient telomere lengths contribute to unlimited self-renewal and genomic stability of pluripotent stem cells (PSCs) [50, 51]. Crotonic acid-induced histone Kcr may protect telomeres by activating two-cell genes and Zscan4 and increasing T-SCE-based ALT-like activity [52]. Moreover, Kcr enhances the efficiency of chemical induction of pluripotent cells [52], although more experiments are needed to understand whether crotonylation directly or indirectly regulates the induction process.

Recently, during differentiation of ESCs, key crotonyl-CoA-producing enzymes such as ACSS2, ACADS, and ACOX3 were significantly induced and enriched in endoderm and/or mesoderm differentiation, indicating endoderm differentiation is associated with increased crotonyl-CoA production [18]. Histone crotonylation and endodermal gene expression were enhanced upon differentiation of endoderm [18]. Furthermore, endoderm differentiation was promoted by crotonate, and disrupted histone crotonylation by deletion of crotonyl-CoA-producing enzymes impaired meso/endoderm differentiation [18].

Most recently, systematic crotonylome profiling in mouse PSCs in different states displayed that majority of crotonylated proteins were involved in pluripotency-related pathways such as RNA biogenesis, central carbon metabolism, and proteasomal degradation [53]. High crotonyl-CoA levels by adding crotonic acid promoted proteasome activities in metastable PSCs and facilitated sustaining of pluripotency [53].

### HIV latency

Epigenetic regulation of histone tails at the human immunodeficiency virus (HIV) long-terminal repeat is essential for the establishment, maintenance, and reactivation of HIV latency [54]. Elevated histone Kcr by ACSS2 at the HIV LTR caused the reactivation of latent HIV and viral transcription [55], suggesting its potential role in HIV latency establishment. Besides, a remarkable synergistic reactivation of latent HIV arises when ACSS2-induced histone Kcr is combined with either PKC agonist PEP005, or vorinostat [55]. Besides, high level of ACSS2 in intestinal mucosa was correlated with altered fatty acid metabolism in the simian immunodeficiency virus-infected non-human primate models of AIDS [55].

### Carcinogenesis

Histone H3K18cr was the most abundant histone Kcr in intestine, especially in the TSS of colon epithelial crypts [14]. SCFAs are the main products of gut microbiota and affect cellular metabolism and gene transcription in intestine. Depletion of the gut microbiota of mice with antibiotics not only led to a drop in luminal and serum SCFAs, but also caused an increased expression of HDAC2 and decline of histone Kcr in colon [14]. Besides, bioinformatics analysis revealed that high level of H3K18cr was involved in cancer [14]. Gut microbiota modulated carcinogenesis via various manners [56], and these above studies suggested that dysregulation of gut microbiota may affect carcinogenesis by altering histone Kcr. Future studies may focus on the regulation mechanism of microbiota, SCFAs, and histone Kcr in modulating carcinogenesis.

Later, crotonylome alterations by p300 were involved in nonsense-mediated decay, infectious disease, and viral/eukaryotic translation pathways [25]. Additionally, 4.5% of the cancer protein biomarkers in the Early Detection Research Network database were crotonylated [25]. 5.9% of total genes in the Catalogue of Somatic Mutations in Cancer cancer gene database were found to encode proteins crotonylated by p300 [25]. Six p300-targeted crotonylated proteins were confirmed as cancer-related proteins [25].

Crotonylated proteins were widely expressed in human tumor tissues [57]. The global Kcr was decreased in liver, stomach, and kidney carcinomas, and elevated in thyroid, esophagus, colon, pancreas, and lung carcinomas [57]. This indicated Kcr may play diverse roles in cancer progression by modulating different pathways. Changes in global Kcr may partially reflect its association with cancer progression; however, more specific and critical crotonylation factors regulating cancer progression are waiting for unearthing.

### Spermatogenesis

An intense labeling of histone Kcr was observed in post-meiotic male germ cells and was related with X-linked haploid cell-specific gene expression program, indicating a role of histone Kcr in epigenetic modification in the post-meiotic stages of spermatogenesis [5]. Besides, the negative regulation of histone Kcr by CDYL contributed to transcriptional repression and affected the reactivation of sex chromosome-linked genes in round spermatids and the genome-wide histone replacement in elongating spermatids [58]. In *Cdyl* transgenic mice, the dysregulation of histone Kcr by *Cdyl* was associated with reduction of male fertility with a decreased epididymal sperm count and sperm cell motility [58], implicating CDYL-regulated histone Kcr alteration played an essential role in spermatogenesis. Most recently, Kcr was significantly enriched at H3K27 compared to Kac during mouse spermatogenesis [59]. Besides, a combined high level of H3K27ac and H3K27cr existed in super-enhancers determined in spermatocytes and round spermatids [59].

### Tissue injury

Histone Kcr levels were increased in mouse kidney tissue during acute kidney injury (AKI) induced by folic acid or cisplatin treatment [60]. The increased histone Kcr in mouse kidney tissue during AKI was associated with increased PGC-1a and SIRT3 and decreased CCL2 [60]. Furthermore, after adding crotonate in cultured tubular cells or intraperitoneal injection of crotonate, high level of Kcr elevated the expression of PGC-1a and SIRT3 and enhanced protection from AKI [60]. Thus, crotonate may have a potential therapeutic effect on kidney damage, specifically in AKI by increasing histone Kcr [60].

### Inflammation

By utilizing the LPS-induced inflammatory response in RAW264.7, histone Kcr was enhanced by supplement with crotonate prior to LPS stimulation [15]. However, knockdown of ACSS2 resulted in decreased histone Kcr and expression of inflammatory genes upon LPS stimulation [15]. The recruitment of YEATS domain protein AF9 to LPS-induced genes was enhanced by crotonate pre-treatment in a YEATS-dependent manner [37]. Knockout of AF9 significantly reduced the crotonate response to LPS stimulation but did not abolish it completely, suggesting other Kcr reader(s) may be also involved in this response [37].

### Cardiovascular diseases

In human cardiac hypertrophy, short-chain enoyl-CoA hydratase (ECHS1) was reduced, which was coupled with elevated H3K18cr and H2BK12cr. Deficiency of ECHS1 markedly increased H3K18cr, H2BK12cr, and NFATc3 levels, which further drove the expression of hypertrophic fetal genes and finally promoted the hypertrophic growth of neonatal cardiomyocytes, indicating the essential role of ECHS1 and histone crotonylation in maintaining the maturity and homeostasis of cardiomyocytes [61].

## The functions of Kcr in plants

After initial identification of Kcr [5], crotonylome analysis in tobacco [7], papaya fruit [62], rice [63], and peanut [64] have been reported (Table 2). In rice, Kcr and Kbu were enriched as histone modification marks that regulate gene expression [65] (Table 2). Under starvation or submergence, Kcr and Kbu displayed less dynamic compared to H3K9ac, indicating these modifications may display distinct responses to external and internal signals and may represent novel epigenetic mechanisms to fine-tune gene expression for plant adaptation [65]. In response to low temperature, temperature-induced lipocalin-1-like (DgTIL1) was crotonylated, which prevented the degradation of nonspecific lipid transfer protein (DgnsLTP). DgnsLTP then promoted expression and activity of POD, which decreased the accumulation of ROS under cold stress and promoted the cold resistance of chrysanthemum [66]. Besides, crotonylome analysis in chrysanthemum at low temperature identified 393 upregulated and 500 downregulated proteins [67]. Furthermore, crotonylated ascorbate peroxidase (APX) increased APX activity and further reduced the oxidative damage caused by low-temperature stress [67] (Table 2). In addition, various crotonylated proteins in tea plants under NH_4_^+^ deficiency/resupply were found to participate in diverse biological processes such as photosynthesis, carbon fixation, and amino acid metabolism [68] (Table 2), suggesting a profound role of Kcr on the metabolic processes in tea leaves.

**Table 2:** Large-scale proteomic studies of Kcr.

Organism	Biological sample analyzed	Number of Kcr sites	Number of Kcr proteins	Biological process or condition studied	Year	Refs
Human						
* H. sapiens*	Hela	28	histone proteins	spermatogenesis	2011	[5]
* H. sapiens*	Hela	558	453 non-histone proteins	Sodium crotonate treatment	2017	[6]
* H. sapiens*	A549	5096	1579 histone and non-histone proteins	SAHA treatment	2017	[8]
* H. sapiens*	H1299	2696	1024 non-histone proteins	NA (global Kcr survey)	2017	[9]
* H. sapiens*	HCT116	816	392 non-histone proteins	P300 knockout	2018	[25]
* H. sapiens*	Hela	8	histone proteins	CDYL knockout	2017	[58]
* H. sapiens*	Hela	14311	3734 non-histone proteins	CDYL knockout	2020	[22]
* H. sapiens*	Peripheral blood	1109	347 non-histone proteins	normal and maintenance hemodialysis patients	2018	[90]
* H. sapiens*	Peripheral blood	770	353 non-histone proteins	normal and patients with immunoglobulin A nephropathy	2020	[91]
Mouse						
* M. musculus*	MEFs	24	histone proteins	spermatogenesis	2011	[5]
* M. musculus*	Liver	10034	2245 non-histone proteins	NA (global Kcr survey)	2020	[92]
Plant						
* O. sativa*	Seedling leaves	45	histone proteins	starvation and submergence treatments	2018	[65]
* O. sativa*	Seedling leaves	1265	690 non-histone proteins	NA (global Kcr survey)	2018	[63]
* N. tabacum L*.	Leaves	2044	637 non- histone proteins	NA (global Kcr survey)	2017	[7]
* A. hypogaea L*.	Leaves	6051	2508 non-histone proteins	NA (global Kcr survey)	2021	[64]
* C. sinensis L*.	Leaves	2288	971 non-histone proteins	NH_4_^+^ deficiency/resupply	2019	[68]
* C. papaya L*.	Papaya fruit	5995	2120 non-histone proteins	NA (global Kcr survey)	2018	[62]
* D. grandiforum*	Leaves	2017	1199 non-histone proteins	Low temperature	2021	[67]
Microbiology						
* S. roseosporus*	Cultured cells	3944	1389 non-histone proteins	ΔprcB/A mutant	2020	[71]
* C. albicans*	Cultured cells	5242	1584 non-histone proteins	NA (global Kcr survey)	2021	[73]
* R. mucilaginosa*	Cultured cells	1691	629 non-histone proteins	patulin treatment	2018	[74]
* S. roseosporus*	Cultured cells	3944	1389 non-histone proteins	proteasome-deficient ΔprcB/A	2020	[71]
*B. cinerea*	Cultured cells	3967	1041 non-histone proteins	NA (global Kcr survey)	2020	[88]
Other species						
* D. rerio*	Embryos	557	218 non-histone proteins	NA (global Kcr survey)	2018	[89]
* E. sinensis*	Testis	2799	908 histone and non-histone proteins	NA (global Kcr survey)	2020	[87]
* T. gondii*	T. gondii RH strain	12152	2719 non-histone proteins	NA (global Kcr survey)	2021	[93]

*H. sapiens* *Homo sapiens*, *M. musculus* *Mus musculus*, *O. sativa* *Oryza sativa*, *N. tabacum L.* *Nicotiana tabacum L.*, *C. sinensis L.* *Camellia sinensis L.*, *C. papaya L.* *Carica papaya L.*, *R. mucilaginosa* *Rhodotorula mucilaginosa*, *S. roseosporus* *Streptomyces roseosporus*, *B. cinerea* *Botrytis cinerea*, *D. rerio* *Danio rerio*, *E. sinensis* *Eriocheir sinensis*, *C. albicans* *Candida albicans*, *S. roseosporus* *Streptomyces roseosporus*, *D. grandiforum* *Dendranthema grandiforum*, *A. hypogaea L.* *Arachis hypogaea L.*, *T. gondii* *Toxoplasma gondii*, MEFs mouse embryonic. fibroblasts

## The functions of Kcr in microbiology

The conserved histone Kcr was detected in yeast *Saccharomyces cerevisiae* [5]. Yeast HATs (Gcn5, Rtt109, and HAT1) and HDACs (Rpd3, Hos1, and Hos2) were identified as crotonyltransferases and decrotonylases for their function in regulating H3K9cr levels [26]. In addition, yeast Yaf9 and Taf14 were found as Kcr readout [26, 37]. During yeast metabolic cycle (YMC), the periodical expression of fatty acid β-oxidation genes was coincident with histone crotonylation. During nutrient limitation, H3K9cr peaked while K3K9ac declined, and expression of pro-growth genes was prohibited [69]. Adding of crotonic acid elevated the Kcr levels and the constitutive repression of pro-growth genes and caused the disruption of YMC oscillation [69]. Yeast Taf14 was necessary for the transcriptional oscillation of YMC [69]. Besides, Yeast Taf14 was participated in PIC stabilization and was required for yeast survival [70].

In *Streptomyces roseosporus*, Kcr upregulated carbon catabolite repression metabolism by negative regulating the activity of glucose kinase Glk and the utilization of carbon sources [71]. Kcr level and Glk activity were modulated by decrotonylase CobB and crotonyltransferase Kct1 [71] (Table 2).

Histone crotonylation in pathogenic *Candida albicans* was dynamically controlled by metabolism and stress responses [72]. Crotonate can regulate responsive transcriptional program and result in resistance against cell wall stress [72]. Taf14 is essential for *Candida albicans* virulence by controlling gene expression, stress resistance and invasive growth via its chromatin reader function [72]. Crotonylome analysis in *Candida albicans* displayed that majority of crotonylated proteins were involved in biosynthetic events and carbon metabolism [73].

After treatment with patulin, 79 upregulated crotonylated proteins were involved in tricarboxylic acid cycle and gluconeogenic pathway and 46 downregulated crotonylated proteins were related with ribosome and carbohydrate transport and metabolism, which predicted the role of Kcr in patulin degradation [74].

## The identification and detection of Kcr

### Experimental methods

Due to the development of high-sensitivity mass spectrometry, new PTMs could be identified. Unbiased, systematic screenings have been applied to discover new lysine acylations [1]. A pan antibody against Kcr was generated to directly detect Kcr [5]. Isotopic labeling, previously used for the detection of Kac [75], was also used to detect Kcr [5]. Xie et al. developed the genetically encoded photoaffinity analogues of Kcr that can site-specifically incorporate into proteins via the genetic code expansion strategy [76]. The crotonyl mark is highly reactive toward phosphine nucleophiles that contain a pendent carboxylic acid group [77]. Based on water-soluble phosphine warhead, a covalent chemo-proteomic probe for the detection and functional analysis of Kcr was developed, allowing detection of endogenous cellular proteins being crotonylated [77]. Most recently, single-step fluorescent probes (KTcr-I that is recognized by Sirt2, and KTcr-II that is recognized by HDAC3), which generate fluorescence signal by intramolecular nucleophilic exchange reaction, to detect decrotonylation activity of HDACs were developed [78].

Although extensive structural and mechanistic studies, the cross regulation between different types of acylations remains unclear. In order to clarify the role of different lysine acylations, development of acyl-type specific enzymes would be a useful tool. For this, p300 I1935G and CBP I1432G mutants with deficient HAT but competent HCT activities [24] and HDAC1/3 AGG-VRPP mutant with lacking of HDCR but intact HDAC activities [16] were generated. Most recently, relying on replacing an essential active-site lysine residue of orotidine-5’-monophosphate decarboxylase with lysine derivatives by genetic code expansion, a selection system for HDAC-HDCR was designed in yeast [79].

### Bioinformatics tools

Experimental approaches for identifying Kcr sites are often time-consuming and labor-intensive, thus difficult to widely popularize in large-scale species. On the other hand, computational approaches are cost-effective and can be used in a high-throughput manner to generate relatively precise identification. A discrete hidden Markov model implemented with a software named CrotPred for predicting Kcr sites was established [80]. Then, a new approach to predict Kcr sites based on support vector machine was presented [81]. To improve the performance of the computational prediction of crotonylation sites, CKSAAP CrotSite was developed [82]. Based on the CKSAAP CrotSite model, whose sensitivity reached 92.45%, a user-friendly web-server was established [82]. In the meanwhile, a user-friendly web-server named iKcr-PseEns by incorporating five tiers of amino acid pairwise couplings into the general pseudo amino acid composition was also built [83]. Malebary et al. proposed an improved Kcr predictor named iCrotoK-PseAAC, in which various position and composition relative features along with statistical moments were incorporated in this predictor [84]. Later, based on physicochemical property and evolutionary-derived feature of protein sequences, LightGBM-CroSite was developed [85]. Most recently, Lv et al. performed a deep learning-based method termed Deep-Kcr by combining sequence-based features, physicochemical property-based features and numerical space-derived information [86].

Although these tools showed powerful prediction, experimental approaches need to be employed to confirm the prediction results. Since experimental approaches are visible to reflect the dynamics of modification, efficient, lab-common, and inexpensive experimental approaches are urgently needed.

### Kcr versus Kac

**Fig. 4:**
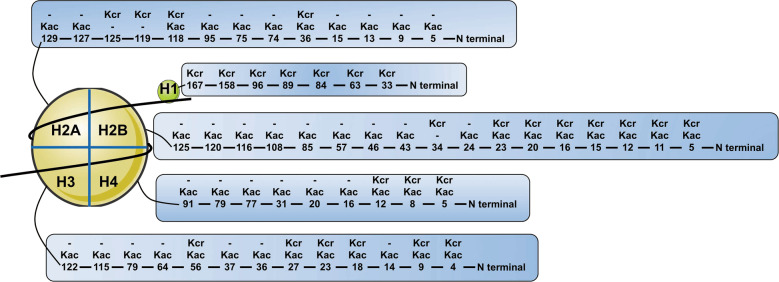
Distribution of lys crotonylation and acetylation on the five human histones. Illustrations of histone Kcr and Kac sites in human cells. Based on PTMs identified in (Tan et al*.* Cell. 2011) [5].

The overlap between histone Kcr and Kac [5] (Fig. 4), raised the possibility of crosstalk between these two PTMs. In Alzheimer’s disease, NEAT1 promoted the autoacetylation of P300 and its acyltransferase activity, and altered the level of H3K27ac and H3K27cr simultaneously [49]. During YMC, both histone crotonylation and acetylation dynamically fluctuated and this fluctuation had distinct peaks at different points in the metabolic cycle [69].

Although Kac and Kcr shared modulators such as writers, erasers, and readers, Kcr may use distinct regulatory modulators from Kac due to the presence of C–C π-bond. Crotonyl group has a more rigid structure. However, the acetyl group is tetrahedral and rotatable. Indeed, YEATS and DPF domain had enhanced binding affinity for Kcr over Kac [37, 38]. Both Kcr and Kac were critical for global transcription in mammalian cells [16]. However, Kcr was reported to preferentially ‘escapee genes’ during post-meiotic sex inactivation in mouse testis [5]. In addition, p300-mediated histone Kcr displayed greater stimulation on gene transcription than histone Kac [15]. CBP/p300 mutants with deficient HAT and intact HCT activity [24] and HDAC1/3 mutants with impaired HDAC but intact HDCR activities [9] indicated different modulation patterns between Kac and Kcr. In distinct metabolic conditions, or different progression status of tissues, the patterns of Kcr and Kac were different, which may be due to the altered concentrations of distinct CoA [5].

### Conclusions and perspectives

Kcr is a recently identified posttranslational modification that occurs in a wide range of proteins both in prokaryotes and eukaryotes [5–7, 26, 65, 71, 72, 87–89] (Table 2). Although Kcr has been shown to be involved in diverse cellular functions in health and disease situations, the underlying mechanism of Kcr in these biological processes are unclear. Aberration in crotonylation and decrotonylation was associated with several diseases. Thus, one of the future focuses may be the in-depth understanding of the substrates targeted by Kcr, and their biological roles regulated by this modification.

Cellular concentration of crotonyl-CoA influenced histone and non-histone Kcr and further altered biological processes [15]. Reports demonstrated that ACSS2 and CDYL regulate the level of crotonyl-CoA in tissues and in cells [15, 22]. Therefore, one angle to clarify the function of Kcr in biological processes is to measure crotonyl-CoA levels in tissues and in subcellular compartments and identify factors that influence the levels of crotonyl-CoA.

The number of enzymes that catalyze or hydrolyze Kcr is still few. In addition, the Kcr writers, erasers, and readers are generally shared with other PTMs and it is unclear whether specific enzymes for Kcr exist. Therefore, identifying these specific enzymes for Kcr would be interesting.

The overlap between Kcr and other PTMs, such as Kcr and Kac [5], aroused consideration on whether different acylations have unique regulatory roles or they perform redundant functions. Besides, investigation into the relative stoichiometries of various acylations occurring on the same lysine residue would be an interesting aspect for future studies.

## Facts

- Lysine crotonylation (Kcr) is newly identified protein posttranslational modification in histone and non-histone proteins, and is involved in diverse diseases and biological processes, such as neuropsychiatric disease, carcinogenesis, spermatogenesis, tissue injury and inflammation, by influencing protein structure and modulate protein stability, localization, and activity.
- The unique carbon–carbon (C–C) π-bond structure of Kcr resulting in a rigid planar conformation indicates distinct regulatory mechanisms from the widely studied other types of lysine acylation.
- The intensity of Kcr could be influenced by the levels of intracellular crotonyl-CoA, the ratio of crotonyl-CoA/acetyl-CoA, as well as the dynamic balance between crotonyltransferase and decrotonylase.
- The functions of Kcr in physiology and pathology are dependent on the readers that recognize Kcr modification. YEATS and DPF domain proteins have been characterized as histone Kcr-preferential reader.
- The overlap between histone Kcr and Kac raises the possibility of crosstalk between these two PTMs that display distinct roles in the same disease and biological process.
- Experimental and computational approaches have been developed for prediction, identification, and analyzing the regulatory mechanisms of Kcr.

## Open questions

- Although Kcr has been shown to be involved in diverse cellular functions in health and disease situations, the underlying mechanism of Kcr roles are unclear. Thus, one of the future focuses may be the in-depth understanding of the substrates targeted by Kcr, and their biological roles regulated by this modification.
- How to measure crotonyl-CoA levels in tissues and in subcellular compartments? What are factors that influence the levels of crotonyl-CoA in tissues and subcellular compartments?
- Whether specific enzymes that catalyze or hydrolyze histone Kcr exist? Whether non-histone Kcr uses distinct crotonyltransferases and decrotonylases from histone Kcr because of their diverse locations?
- Whether readout of non-histone kcr shares similar recognition mechanisms as histone Kcr?
- The overlap between Kcr and other PTMs, such as Kcr and Kac, arouses consideration on whether different acylations have unique regulatory roles or they perform redundant functions. Besides, investigation into the relative stoichiometries of various acylations occurring on the same lysine residue would be an interesting aspect for future studies.
- The efficient, lab-common, and inexpensive experimental approaches for Kcr detection are urgently needed. For clarifying the roles of different lysine acylations, development of acyl-type specific enzymes would be helpful.

## Supplementary information

Supplementary figure legends

Supplementary Figure 1

Supplementary Figure 2

Supplementary Figure 3
