# Supplementary material for: Protein lysine crotonylation: past, present, perspective
Source: Cell Death Dis. 2021 Jul 14;12(7):703. doi: 10.1038/s41419-021-03987-z (PMC8280118; doi:10.1038/s41419-021-03987-z)
Supplement: Supplementary file 1 — Supplementary figure legends [file 41419_2021_3987_MOESM1_ESM.docx]

**Protein Lysine Crotonylation: Past, Present, Perspective**

Gaoyue Jiang^1, 3^, Chunxia Li^2, 3^, Meng Lu^2, 3^, Kefeng Lu^2, *^, and Huihui Li^1, *^

^1^West China Second University Hospital, State Key Laboratory of Biotherapy, and Key Laboratory of Birth Defects and Related Diseases of Women and Children, Ministry of Education, Sichuan University, 610041 Chengdu, China

^2^Department of Neurosurgery, State Key Laboratory of Biotherapy, West China Hospital, Sichuan University and The Research Units of West China, Chinese Academy of Medical Sciences, China

^3^These authors contributed equally

^*^Correspondence: Kefeng Lu (lukf@scu.edu.cn) and Huihui Li (lihuihui@scu.edu.cn)

**Running title**

Regulation, readout and physiological functions of Kcr

**Supplementary Figure 1** Domain organization of histone acetyltransferases (HATs) (writers).

**Supplementary Figure 2** Domain architectures of histone deacetylases (HDACs) (erasers).

**Supplementary Figure 3** Domain organization of lysine acetylation recognizers (readers).

**Supplementary Figure Legends**

**Supplementary Figure 1 Domain organization of histone acetyltransferases (HATs) (writers).**

Domain organization of representative histone acetyltransferases (HATs) (writers). The name and the length of the selected proteins are shown on the bar chart. Red star represents that the proteins have been reported as HCTs that use crotonyl-CoA as substrate to catalyze Kcr. Three major HAT families including p300/CREB-binding protein (p300/CBP), MYST, and GNAT (Gcn5-related N-acetyltrasferase) were characterized by their sequences and structures^1^.

**Supplementary Figure 2** **Domain architectures of histone deacetylases (HDACs) (erasers).**

Domain architectures of histone deacetylases (HDACs) (erasers). The name and the length of the selected proteins are shown on the bar chart. Red star represents that the proteins have been reported as HDCRs that function as eraser of lysine crotonylation^2^. There are four groups of histone deacetylases (HDACs): Class I (HDAC1, 2, 3, and 8), II (HDAC4, 5, 6, 7, 9, and 10), III (SIRT1-7) and IV (HDAC11)^3^. It has been shown that class I, II, and IV HDACs are Zn^2+^-dependent histone deacetylases (Zn^2+^-dependent HDACs) and class III HDACs are NAD^+^-dependent sirtuins.

**Supplementary Figure 3** **Domain organization of lysine acetylation recognizers (readers).**

Domain organization of representative lysine acetylation recognizers (readers). The name and the length of the selected proteins are shown on the bar chart. Red star represents that the proteins have been reported as Kcr readers that can recognize Kcr^4, 5^. The level of Kcr could be influenced by the levels of intracellular crotonyl-CoA, and the ratio of crotonyl-CoA/acetyl-CoA, as well as the dynamic balance between crotonyltransferase and decrotonylase^2^ . Thus, the function of Kcr modification in physiology and pathology may be dependent on the readers that recognize Kcr modification. For the well-studied histone Kac, three major families of readers have been characterized: bromodomain proteins, YEATS ( ENL, AF9, and YEATS2) domain proteins, and double plant homeodomain finger (DPF) proteins^2^.

**References:**

1. Sabari BR, Zhang D, Allis CD, Zhao Y. Metabolic regulation of gene expression through histone acylations*. Nat Rev Mol Cell Biol* **2**, 90-101 (2017).

2. Zhao S, Zhang X, Li H. Beyond histone acetylation—writing and erasing histone acylations*. Current Opinion in Structural Biology* **53**, 169-77 (2018).

3. Seto E, Yoshida M. Erasers of Histone Acetylation: The Histone Deacetylase Enzymes*. Cold Spring Harbor Perspectives in Biology* **6**, a18713 (2014).

4. Li Y, Sabari BR, Panchenko T, Wen H, Zhao D, Guan H, *et al*. Molecular Coupling of Histone Crotonylation and Active Transcription by AF9 YEATS Domain*. Molecular Cell* **62**, 181-93 (2016).

5. Xiong X, Panchenko T, Yang S, Zhao S, Yan P, Zhang W, *et al*. Selective recognition of histone crotonylation by double pHd fingers of MOZ and dpF2*. Nature Chemical Biology* **12**, 1111-8 (2016).

6. Jenuwein T. Translating the Histone Code*. Science (American Association for the Advancement of Science)* **293**, 1074-80 (2001).

**校对报告**

当前使用的样式是 [Vancouver New]

当前文档包含的题录共7条

有0条题录存在必填字段内容缺失的问题

所有题录的数据正常
