# Supplementary figures and images for: Protein lysine crotonylation: past, present, perspective
Source: Cell Death Dis. 2021 Jul 14;12(7):703. doi: 10.1038/s41419-021-03987-z (PMC8280118; doi:10.1038/s41419-021-03987-z)

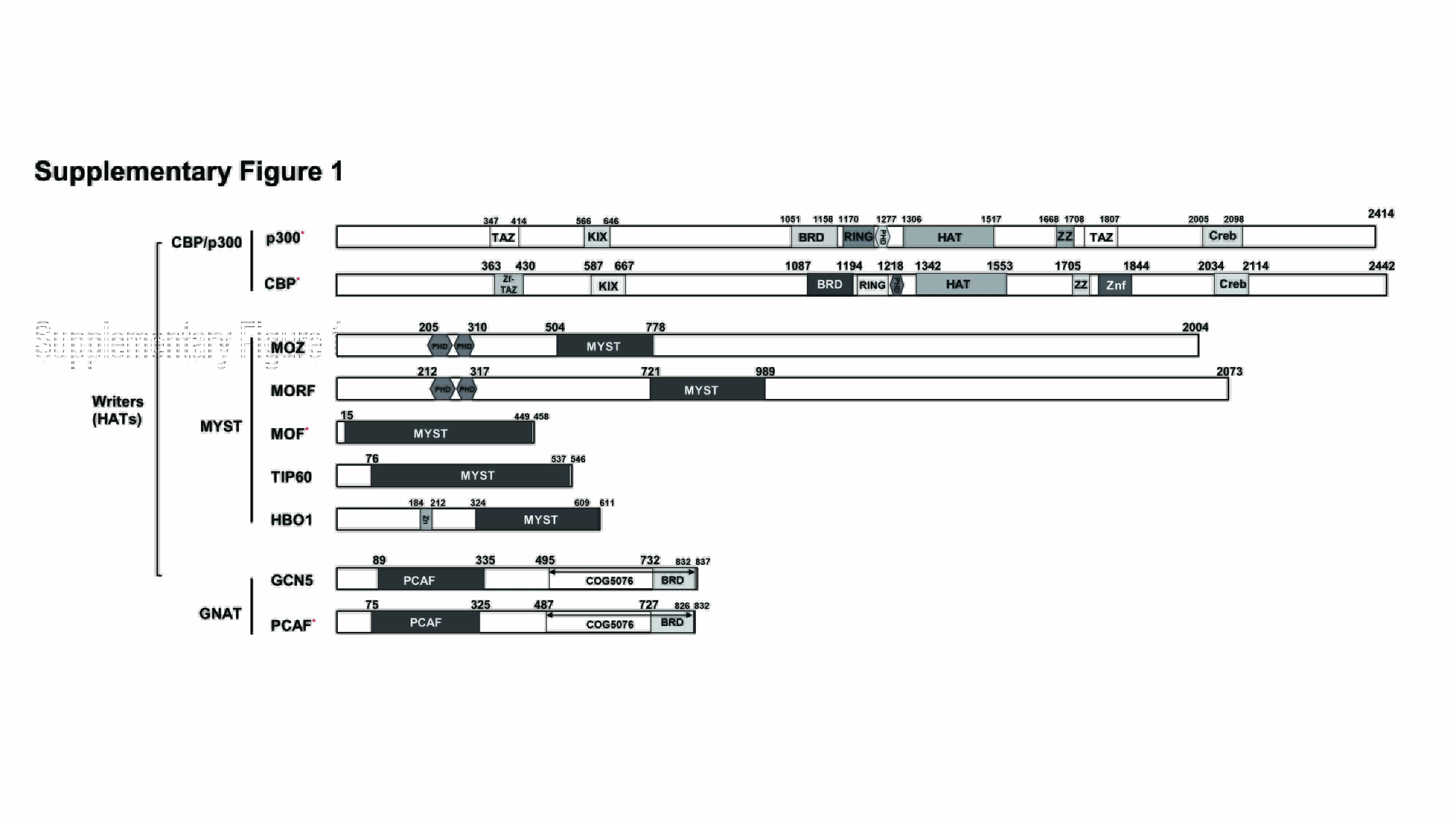

Supplement: Supplementary file 2 — Supplementary Figure 1 [file 41419_2021_3987_MOESM2_ESM.jpg]

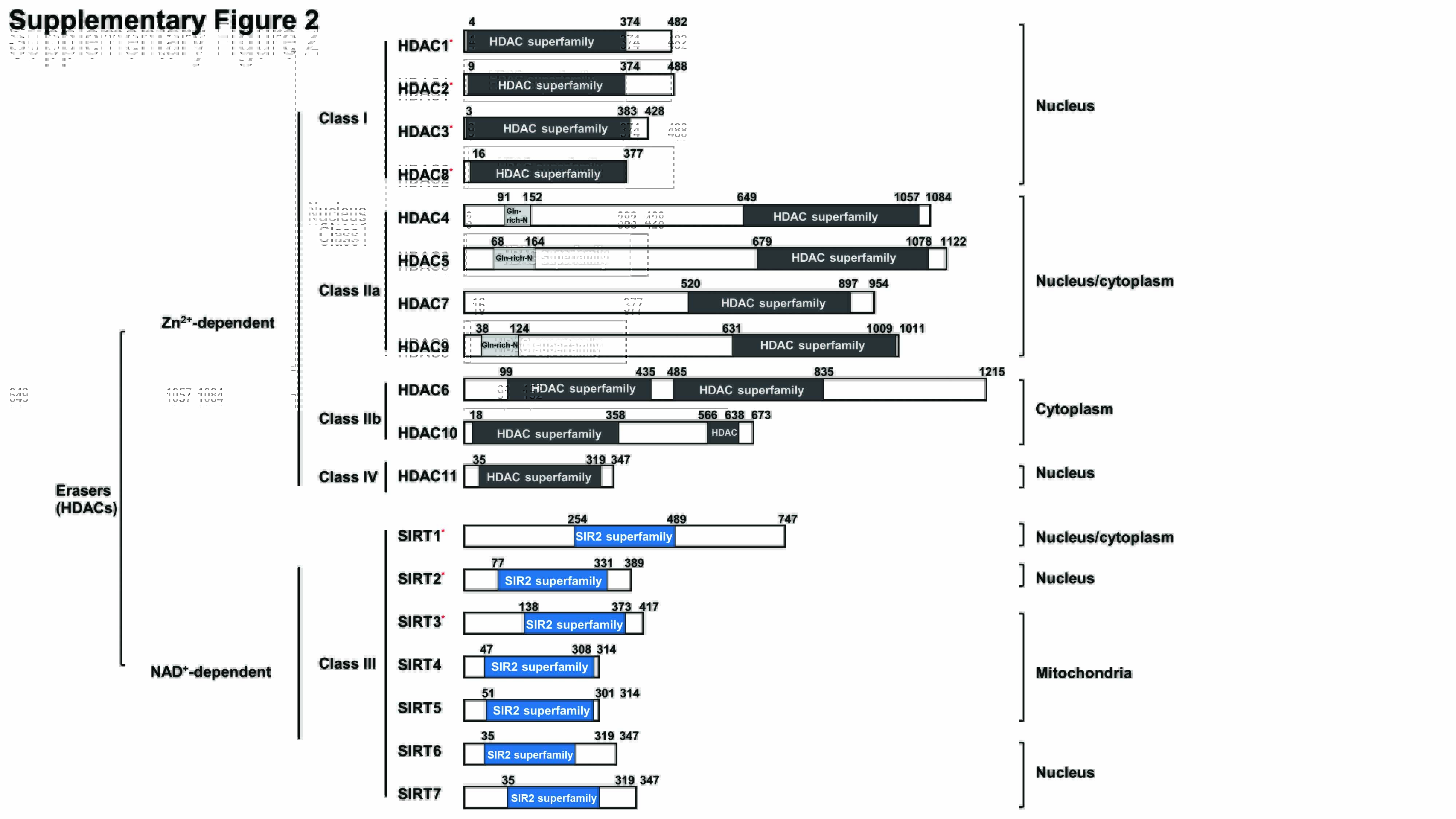

Supplement: Supplementary file 3 — Supplementary Figure 2 [file 41419_2021_3987_MOESM3_ESM.jpg]

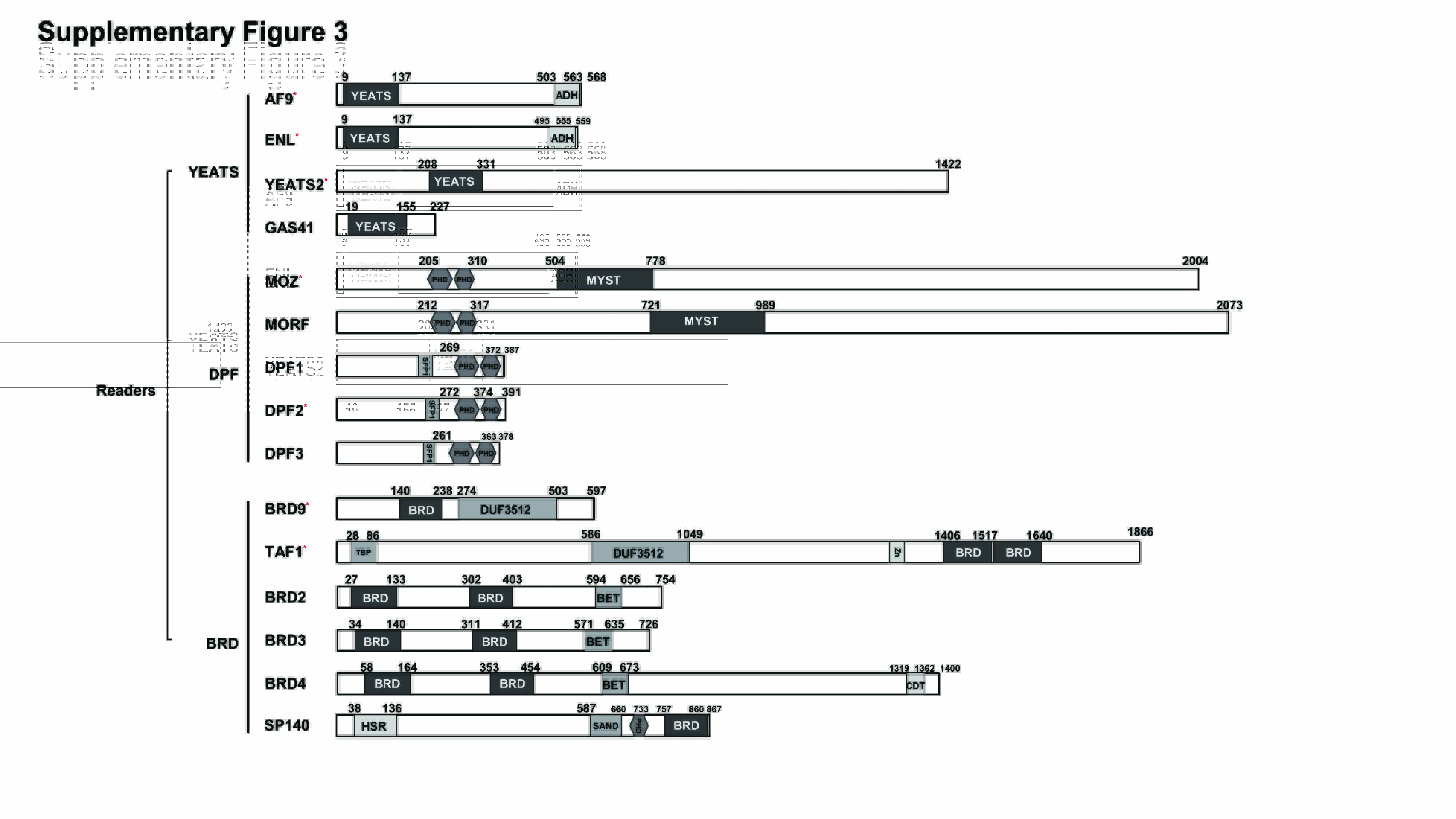

Supplement: Supplementary file 4 — Supplementary Figure 3 [file 41419_2021_3987_MOESM4_ESM.jpg]
